# Supplementary material for: Content-rich biological network constructed by mining PubMed abstracts
Source: BMC Bioinformatics. 2004 Oct 8;5:147. doi: 10.1186/1471-2105-5-147 (PMC528731; doi:10.1186/1471-2105-5-147)
Supplement: Additional File 2 — The original results of the above study (non-essential files are deleted to keep the file size under the limit set by BMC bioinformatics). [file 1471-2105-5-147-S2.bz2 › chilibotAdditionalFile2/dip05/228ID7954478E745/html/CTNNB1_CDH1.html]

 


 **CTNNB1** and **CDH1** 
  
Found 952 abstracts in PubMed, retrieved 05.  
 

 What does Google say? 
 PDF only 
| .edu only 

---

**Interactive relationship** (e.g. stimulation, inhibition, etc)

**Non-interactive relationship** (e.g. studied together, co-existance, homology, etc.)

- Expression of E cadherin  [ **CDH1** ] , alpha catenin  [ **CTNNB1** ] , and beta catenin  [ **CTNNB1** ]  in the process of lymph node metastasis in oral squamous cell carcinoma.  Ref: 12888830 Br J Cancer, 2003
- We have studied the effects of the organophosphorous pesticide malathion on cell viability, actin cytoskeleton, cell adhesion complex E cadherin  [ **CDH1** ]  beta catenin  [ **CTNNB1** ] , and Rho and Rac1 GTPases from the human mammary carcinoma cell line MCF 7.  Ref: 12888906 Int J Oncol, 2003
- AIMS To evaluate E cadherin  [ **CDH1** ] , beta catenin  [ **CTNNB1** ] , gamma catenin  [ **CTNNB1** ] , and ezrin expression and its relation to histopathological features of primary and metastatic Wilms s tumours.  Ref: 12890743 Mol Pathol, 2003
- Significantly greater reduction in expression levels of E cadherin  [ **CDH1** ] , alpha catenin  [ **CTNNB1** ]  and beta catenin  [ **CTNNB1** ]  was found in the metastatic group n=64 compared to the nonmetastatic group n=95 P=0.00.  Ref: 12888830 Br J Cancer, 2003
- BACKGROUND The E cadherin  [ **CDH1** ]  catenin  [ **CTNNB1** ]  adhesion complex is crucial for intercellular adhesiveness and maintenance of tissue architecture.  Ref: 12890743 Mol Pathol, 2003
- CONCLUSIONS Compared with primary tumours, metastatic tumours showed lower expression of E cadherin  [ **CDH1** ]  and gamma catenin  [ **CTNNB1** ] , with nuclear staining for beta catenin  [ **CTNNB1** ] .  Ref: 12890743 Mol Pathol, 2003
- In this study, we screened liver tumors induced by 2 amino dimethylimidazo f quinoline MeIQ in CDF 1 mice for beta catenin  [ **CTNNB1** ]  and APC mutations and other genetic alterations shown to occur in human hepatocellular carcinomas HCC, including mutations in the p53 and H ras genes, c myc amplification and E cadherin  [ **CDH1** ]  promoter methylation.  Ref: 12893427 Cancer Lett, 2003
- These changes were related to a decrease in the expression of the adhesion molecules, E cadherin  [ **CDH1** ]  and beta catenin  [ **CTNNB1** ] , and to the distribution and reactivity of actin microfilaments to TRITC phalloidin.  Ref: 12888906 Int J Oncol, 2003
- Western blotting was used to determine polypeptide size and expression of E cadherin  [ **CDH1** ]  and beta catenin  [ **CTNNB1** ]  in Wilms s tumours compared with normal kidney.  Ref: 12890743 Mol Pathol, 2003
- METHODS Immunohistochemistry was used to determine the expression and cellular distribution of E cadherin  [ **CDH1** ] , beta catenin  [ **CTNNB1** ] , gamma catenin  [ **CTNNB1** ] , and ezrin in primary and metastatic Wilms s tumours.  Ref: 12890743 Mol Pathol, 2003
- These data suggest that evaluation of the immunohistochemical expression of E cadherin  [ **CDH1** ] , alpha catenin  [ **CTNNB1** ]  and beta catenin  [ **CTNNB1** ]  is extremely valuable for the diagnosis of metastatic occurrence.British Journal of Cancer 2003 8 57 563.  Ref: 12888830 Br J Cancer, 2003
- We investigated immunohistochemical expression of E cadherin  [ **CDH1** ] , alpha catenin  [ **CTNNB1** ]  and beta catenin  [ **CTNNB1** ]  in 159 tissue samples from patients with oral squamous cell carcinoma and examined the correlation between their expressions and the presence of regional lymph node metastasis.  Ref: 12888830 Br J Cancer, 2003
- Differential expression of E cadherin  [ **CDH1** ]  and beta catenin  [ **CTNNB1** ]  in primary and metastatic Wilms s tumours.  Ref: 12890743 Mol Pathol, 2003
- In the metastatic process, reduction in cell to cell adhesion including E cadherin  [ **CDH1** ]  catenin  [ **CTNNB1** ]  cell adhesion complex is an essential step.  Ref: 12888830 Br J Cancer, 2003
